# Supplementary material for: 4K-memristor analog-grade passive crossbar circuit
Source: Nat Commun. 2021 Aug 31;12:5198. doi: 10.1038/s41467-021-25455-0 (PMC8408216; doi:10.1038/s41467-021-25455-0)
Supplement: Supplementary file 1 — Supplementary Information [file 41467_2021_25455_MOESM1_ESM.pdf]

Supplementary Information

4K-Memristor Analog-Grade Passive Crossbar Circuit

H. Kim, M. R. Mahmoodi, H. Nili, and D. B. Strukov

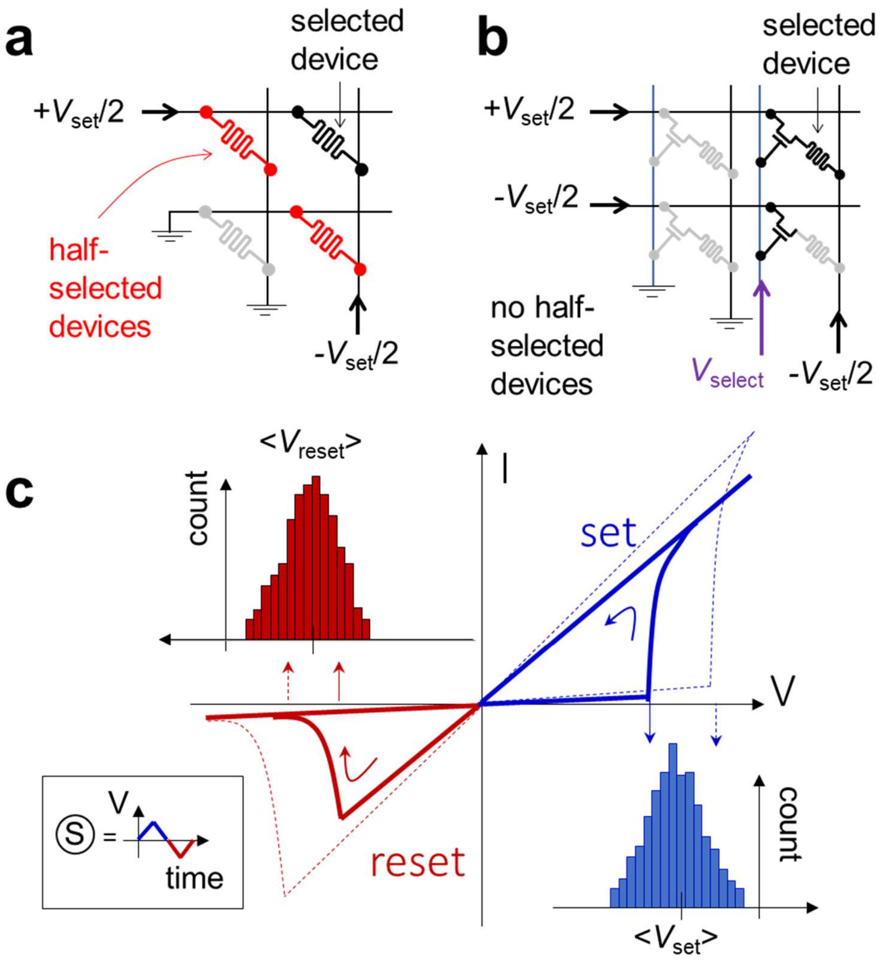

Supplementary Figure 1. Half-select disturbance. A typical half-biasing scheme in (a) passive ("0T1R") and (b) active ("1T1R") crossbar circuits that are employed for applying write voltages to the selected memristor. In 0T1R arrays, a fraction of the external voltage, which is applied to the crossbar array to write the selected device, is dropped across the half-selected devices that share the same horizontal and vertical electrodes with the selected device. On the other hand, the select transistors and additional lines controlling it allow applying a nonzero voltage across the selected device only in the 1T1R circuits. (c) Schematic drawings of representative *I-V* curve and device-to-device distributions for set and reset switching voltages (bottom right and top left insets, correspondingly) which highlight the issue of half-select disturbance in passive crossbar circuits. Specifically, writing the selected device with the switching threshold at the higher end of the distribution can disturb the half-selected devices at the lower end of the distribution if the distribution is wide enough. Adapted from Ref. 1.

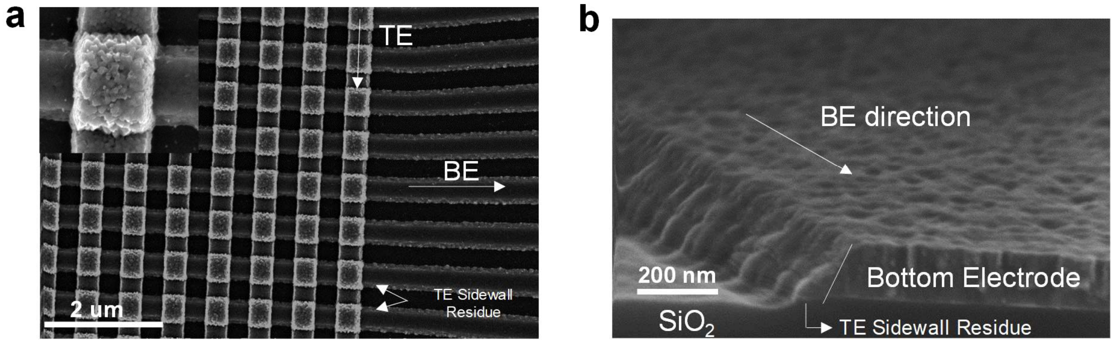

Supplementary Figure 2. Sidewall residue challenge. (a) A top and (b) cross-sectional scanning electron microscopy (SEM) images of the crossbar circuit fabricated without planarization steps. Panel a inset shows a zoomed-in image of a crosspoint area. When the top electrodes (TEs) are patterned with the etching process without the planarization step, a sidewall residue along the bottom electrodes (BEs) results in the shortening of all TEs.

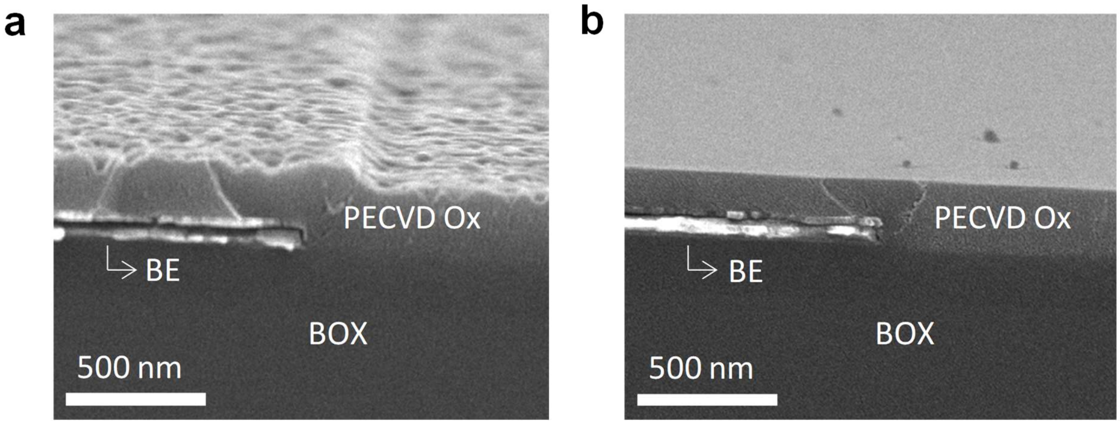

Supplementary Figure 3. Chemical-mechanical polishing calibration. Chemical-mechanical polishing with 80 rpm plate rotate rate, 50 ml/min slurry flow rate under two different conditions for back-pressure: (a) 25 psi and (b) 35 psi. The latter conditions result in higher quality surface and are utilized in device fabrication.

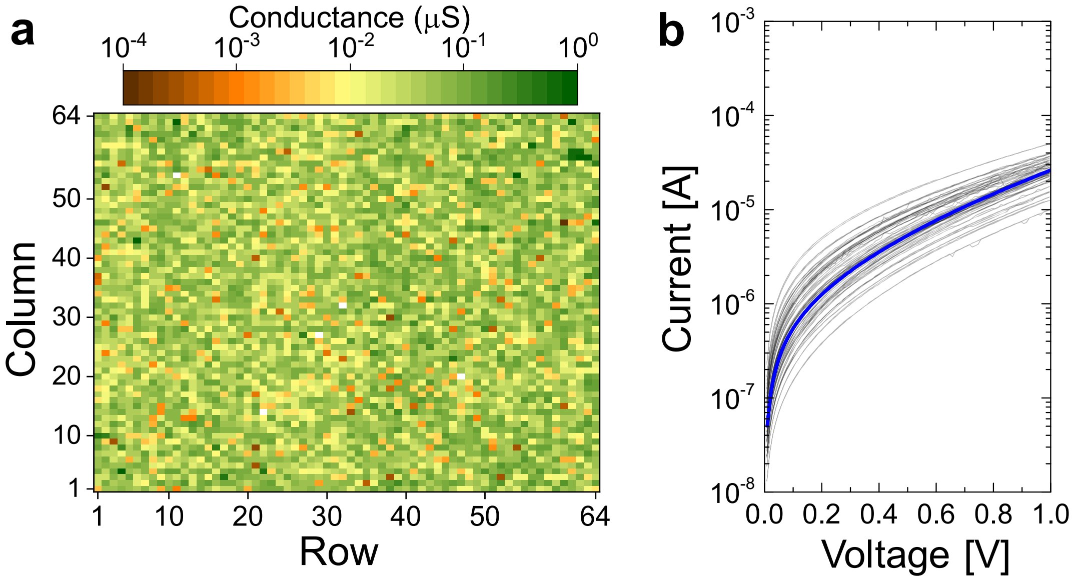

Supplementary Figure 4. As-fabricated crossbar results. (a) The conductance map measured at 0.4 V. Median conductance is ~ 45 nS. (b) *I-V* characteristics for the 36 virgin-state (i.e., before forming) devices of the 6×6 subarray located in the center of the crossbar circuit. The blue curve corresponds to the device highlighted in Fig. 2a of the main text.

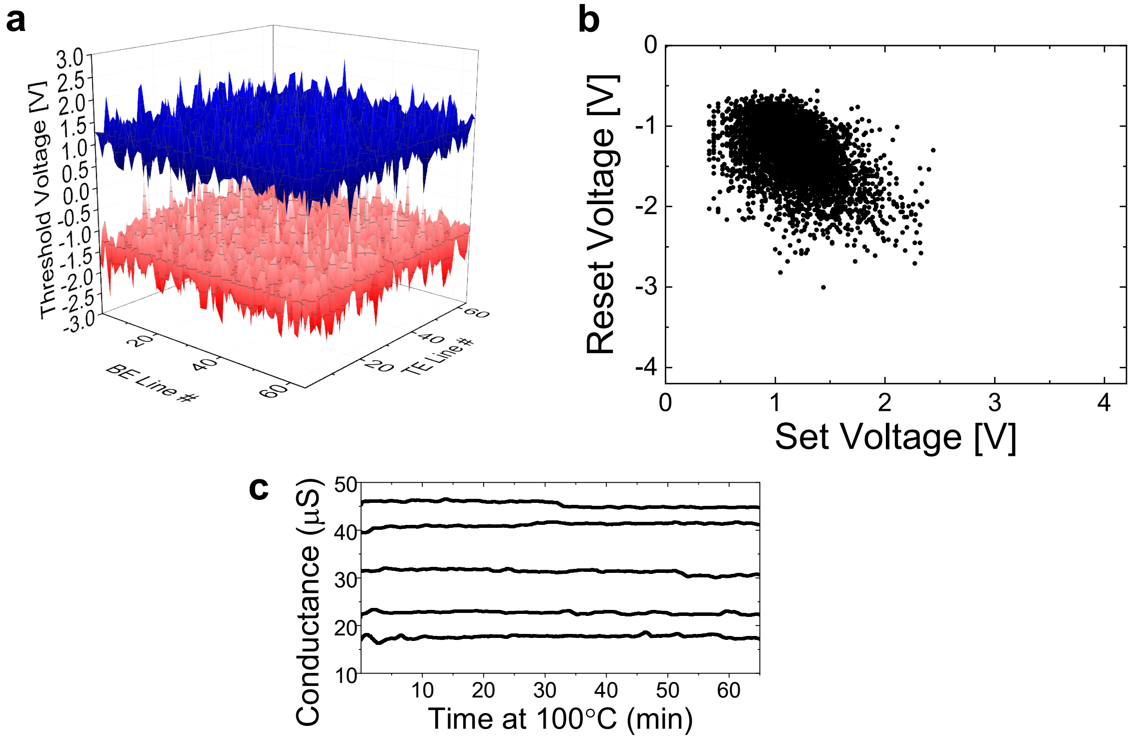

Supplementary Figure 5. Additional crossbar circuit characterization data. (a) Switching threshold voltage map. Blue and red data points correspond to set and reset voltages, respectively. (b) Correlations in switching voltages. The data are post-processed from Fig. 2e of the main text. (c) A retention test is performed after the 1M-cycle endurance test shown in Fig. 2d of the main text. Conductance is measured at 0.1 V at 2 s intervals while continuously baking the crossbar circuit at 100°C.

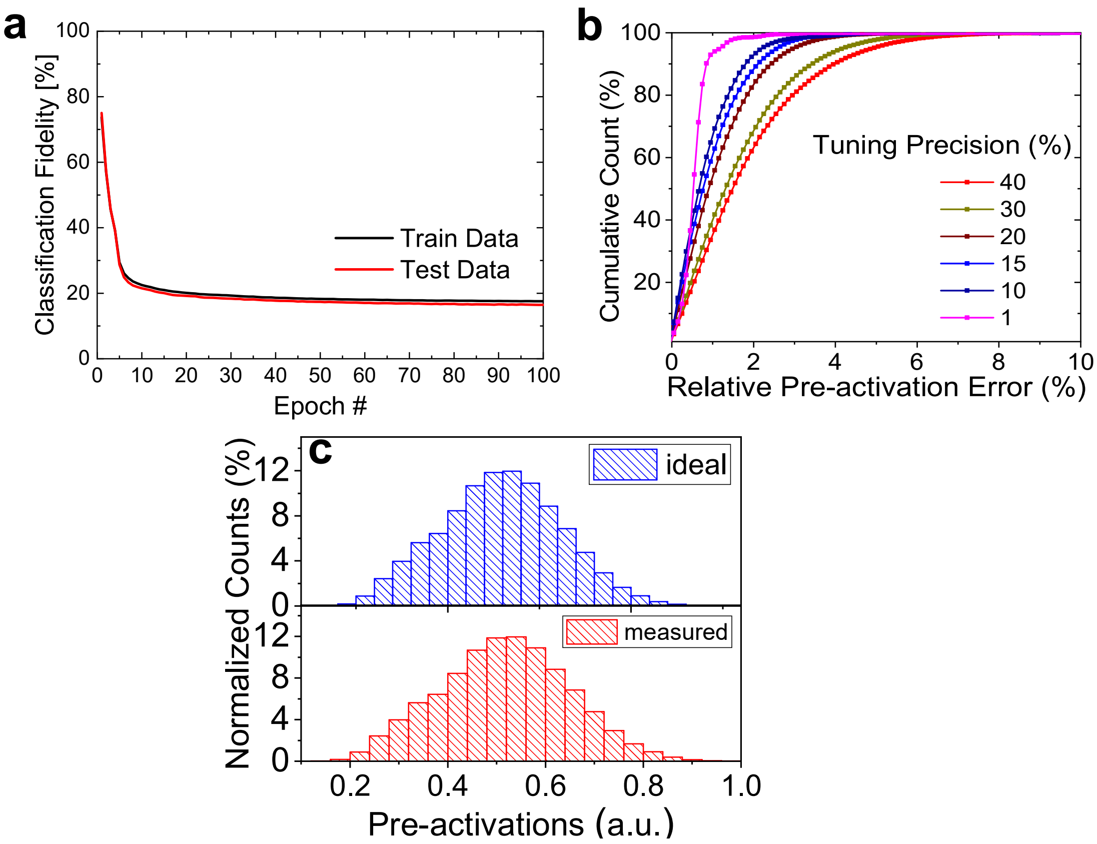

Supplementary Figure 6. Additional data for the classifier experiment. (a) Software-based training results for single-layer perceptron classifier. (b) The cumulative distribution of absolute pre-activation error, i.e., $|I_{\text{ideal}} - I_{\text{measured}}|/(I_{\text{ideal}})_{\max}$, for several studied tuning precisions. The data are computed based on experimentally measured currents and their desired values computed in software for 10^4 test patterns. (c) The comparison of measured and ideal pre-activation distributions for the case of 1% relative tuning precision for 10^4 test patterns.

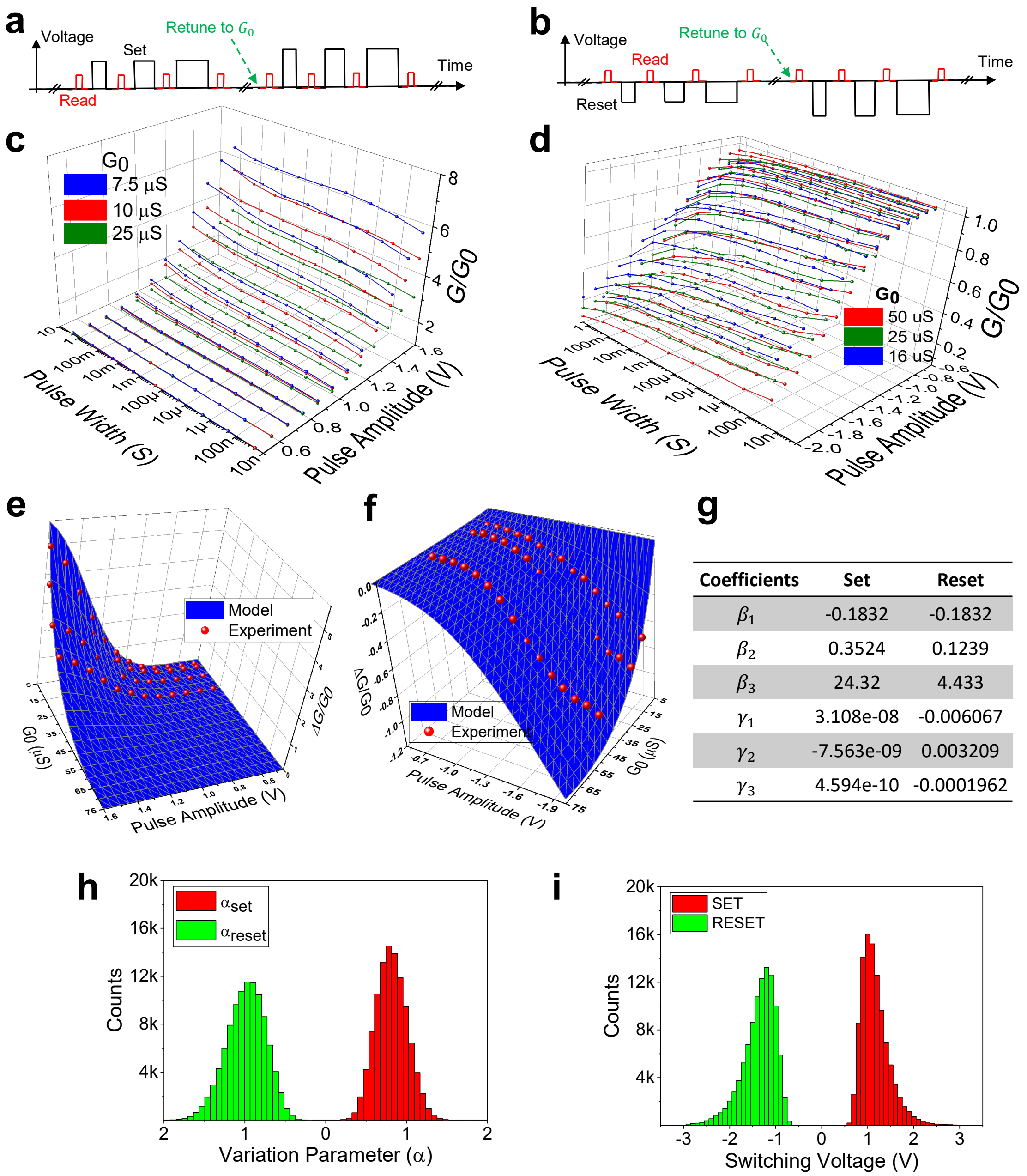

Coefficients	Set	Reset
β_1	-0.1832	-0.1832
β_2	0.3524	0.1239
β_3	24.32	4.433
γ_1	3.108e-08	-0.006067
γ_2	-7.563e-09	0.003209
γ_3	4.594e-10	-0.0001962

Supplementary Figure 7. Modeling half-select disturbance. (a-d) Details of the utilized measurement protocol for modeling (a) set and (b) reset transitions and (c, d) results for conductance changes for three studied cases of initial conductance G_0. Each line with connected dots corresponds to the evolution of the conductance change, normalized to the specific tuned initial value G_0 and averaged across 500 devices, upon application of the voltage pulses with a specific amplitude and exponentially increased duration. (e-i) A phenomenological model for dynamic behavior. The results of fitting dynamic equations to the experimental (e) set and (f) reset data, averaged over 500 devices, and (g) the corresponding model parameters. (h) The distribution of parameter α fitted to reproduce experimentally observed device-to-device variations in Fig. 2f, and (i) predicted by the model variations in the switching threshold for 4096 devices in the modeled 64×64 crossbar circuit. See Supplementary Note 1 for more details. All conductances are specified at 0.1 V.

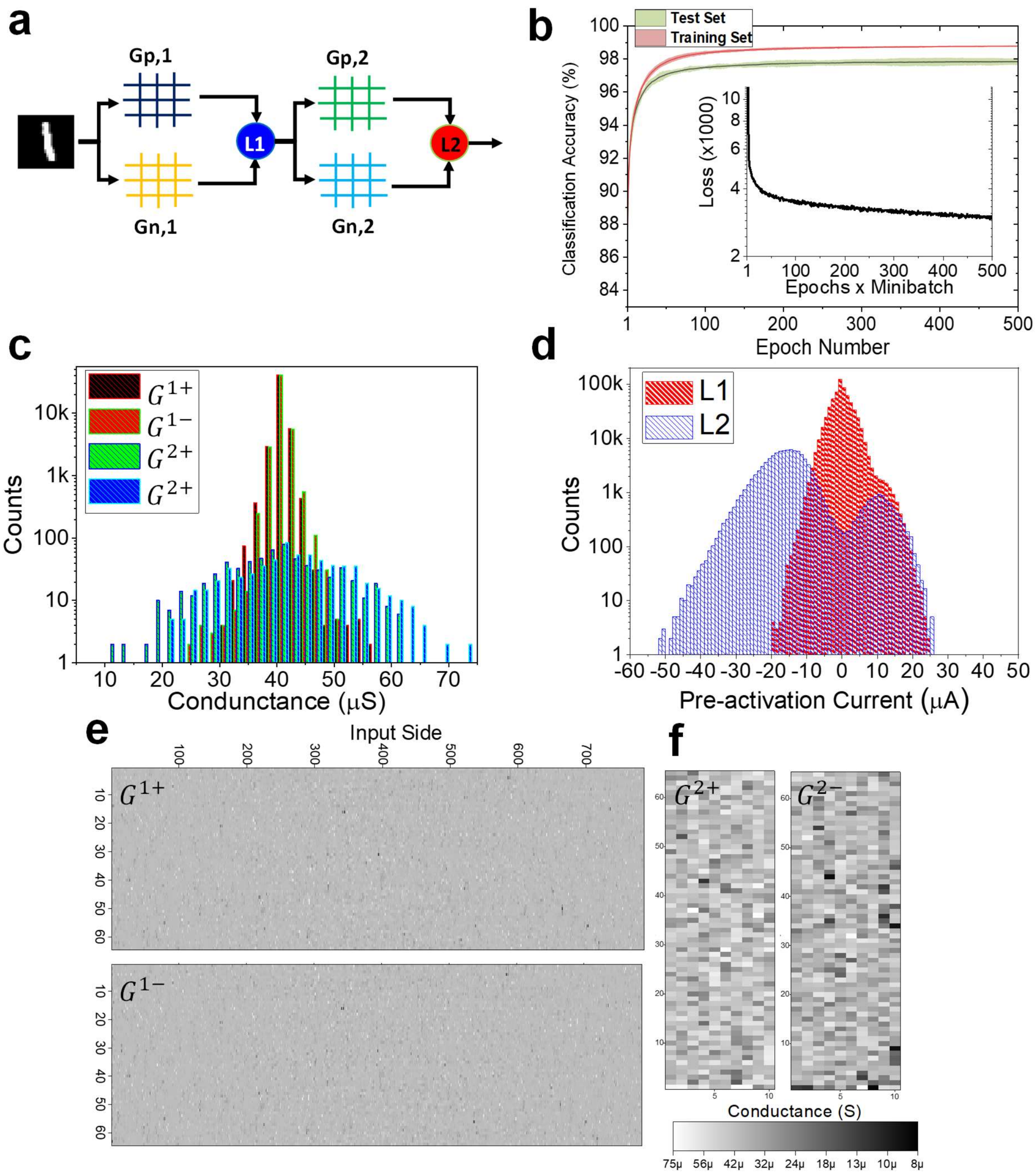

Supplementary Figure 8. Modeling MLP classifier. (a) General scheme for the modeled differential MLP network. (b) The evolution of classification accuracy and cross-entropy loss (inset) during ex-situ training. (c, d) Histograms of (c) the ideal conductances in the 1st positive (G^{1+}), 1st negative (G^{1-}), 2nd positive (G^{2+}), and 2nd negative (G^{2-}) weight layers, and (d) the ideal pre-activation currents for the hidden (L1) and the output (L2) neurons. (e, f) Map of ideal conductances for (e) the 1st layer and (f) the 2nd layer of the network.

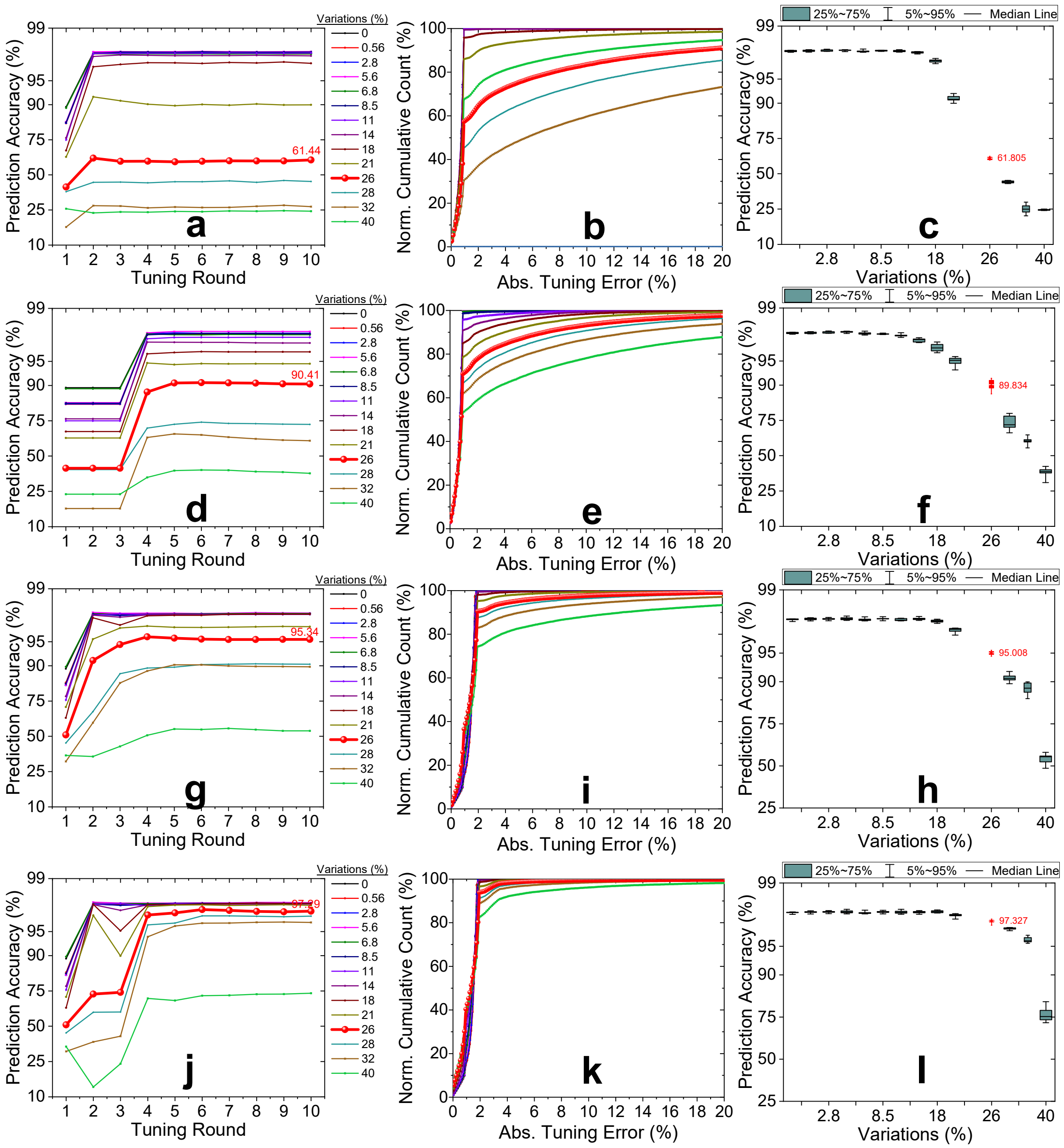

Supplementary Figure 9. Device uniformity impact on MLP accuracy. (a-l) Modeling results when using (a-c) baseline, (d-f) 1st, (g-h) 2nd, and (j-l) 3rd tuning approaches. (a, d, g, j) The improvements in classification accuracy with more rounds of tuning. (b, e, i, k) Cumulative distribution of the absolute tuning error at the end of the 10th tuning round. (c, f, h, l) Classification accuracy as a function of device variations at the end of the 10th tuning round. The box plot shows the statistics over 10 different cases of initial conductances. The thick red lines correspond to the demonstrated technology, i.e., α = ~26%. For simplicity, memristors' static *I-V* nonlinearities and noise are neglected, and ideal peripheral circuits are assumed in simulations. In panel a, the accuracy saturates after a few rounds because of the significant half-select disturbance when re-tuning higher switching threshold devices. In panel d, the utilized maximum values for set / reset thresholds are NA, 2, 0, 1.65, 1.45, 1.4, 1.35, 1.3, 1.2, 1.1 / NA, 0, -1.65, -1.45, -1.35, -1.3, -1.2, -1.1 for tuning rounds #1, #2, …, #10, respectively. In panel j, the highest accuracy is 97.29%.

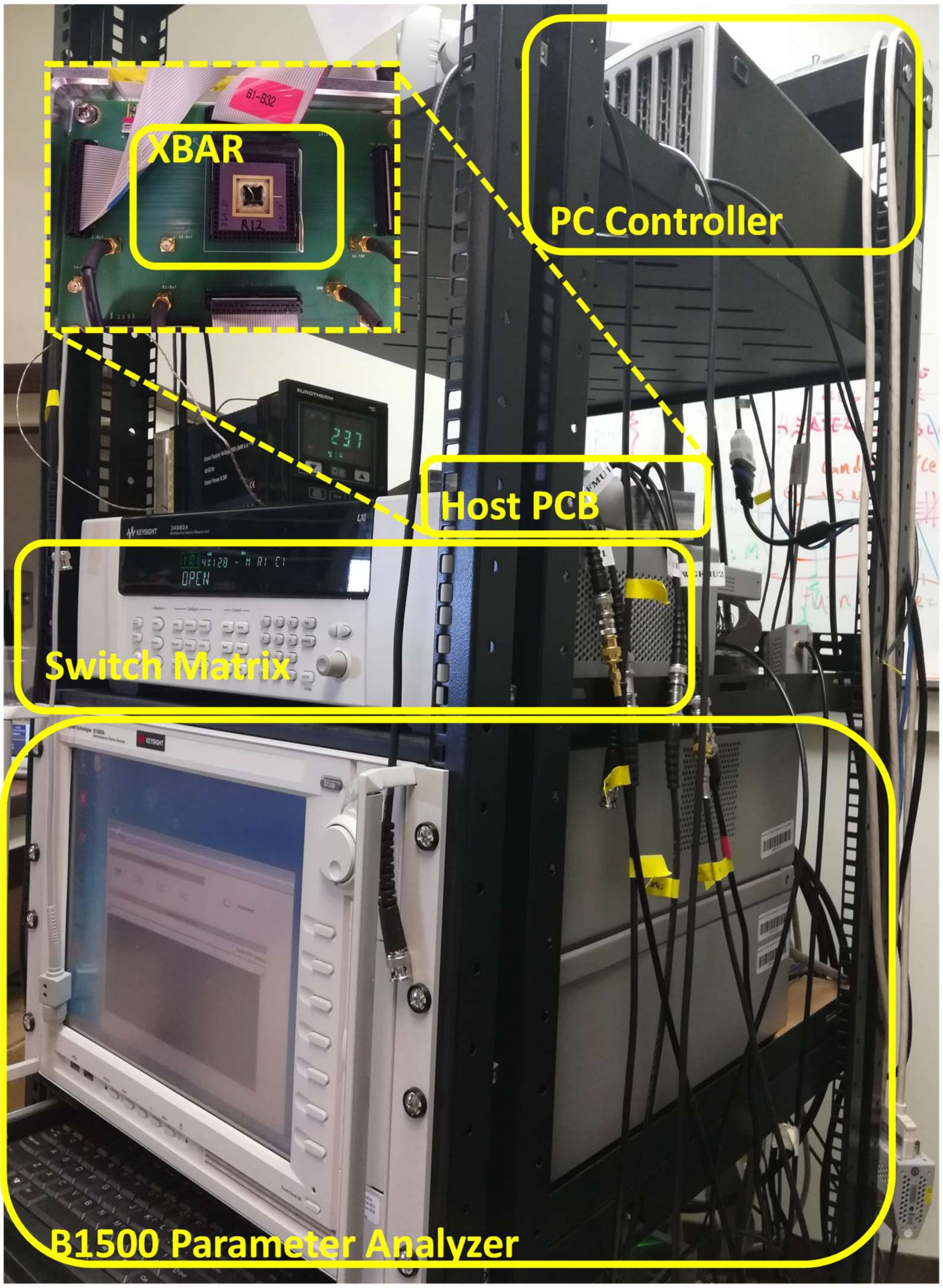

Supplementary Figure 10. Experimental setup. The photo shows the setup's main parts, namely the packaged crossbar circuit mounted on a custom printed circuit board, personal computer controller, Agilent switch matrix, and Agilent B1500 semiconductor device analyzer.

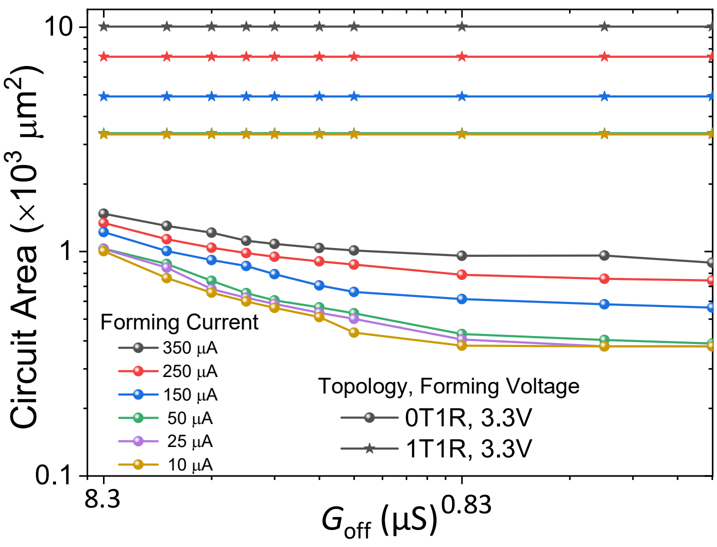

Supplementary Figure 11. Crossbar circuit area scaling with device current. 64×64 crossbar circuit and its peripheral analog muxes areas for two topologies implemented in 65 nm process as a function of leakage and forming/write currents. (For simplicity, the area of sensing circuitry, decoders, and level-shifters are excluded in this figure to avoid performing more comprehensive modeling of scaling down G_{on} and finding optimal design of other peripheral components.) The cell area for 0T1R technology is 250×250 nm^2, which was determined from the layout in the considered CMOS process. Thick-oxide 3.3 V transistors are used to implement select transistors and forming/switching current passing muxes in peripheral circuitry. Their sizing is chosen such as not to exceed the 0.7 V voltage drop and hence limit the maximum input voltage to 4 V. For 0T1R crossbar circuits, an analog switch are designed to pass the forming current to the selected device and leakages through other off-state devices in the crossbar. The leakage currents are modeled assuming negligible line resistance and floating forming configuration [29]. For 1T1R crossbar circuits, an analog switch in the periphery is designed to pass only the forming current though there is less headroom because part of the voltage is also dropped on the selector. The memory and peripheral circuits are assumed to overlap for 0T1R implementation. Note that forming current of 250 μA and G_{off} = 1.056 μS correspond to the memory technology assumptions of the last two columns in Table S3, e.g. max[A, 128B] ≈ 820 μm^2 and A+128B ≈ 7400 μm^2 for 0T1R and 1T1R, correspondingly, where A is an area of the 64×64 crossbar memory array and B is an area of analog mux circuitry serving one line. The simulation results show that for the 1T1R case, the total area is dominated by the cells' select transistor, which is scaled down with lowering forming currents. For the 0T1R case, the total area is due to peripheral muxes, which is reduced at higher G_{off} when lowering leakage currents, but is mostly limited by forming currents at lower G_{off}.

Supplementary Table 1. **Comparison of memristive circuits**. The specific focus of the table is on the state-of-the-art non-volatile (filamentary) analog-grade 0T1R metal-oxide devices, while only few representative works are listed for metal-oxide 1T1R and solid-state-electrolyte 0T1R circuits. Furthermore, the table does not include recent results based on dense commercial "binary" 1T1R technology. Also, note that the common concern for the solid-state electrolyte type devices (rows #1 to #3) and interfacial switching WO_x devices (rows #4 to #6) is poor state retention.

Cell type		Ref.	Crossbar size[0]	Yield (%)	Largest working demo[0]	Cell size[1] (μm^2)	Forming[2] current (μA)/ Voltage(V)	Endur-ance[3] (cycles)	Array level tuning precision	Set switching statistics μ / σ (V)	G_{max} / G_{min}[4] (μS)	Retention (@°C)	Type of integration / patterning technique / Substantial CMOS foundry integration challenges[5]
0T1R	Si/Ag	[2]	32×32	~100	32×32	~1200[8]	5000/3.7	>10M	-	2.25/ 0.1	10 / 1[9]	~hours	SA/RIE/High-T epitaxy&Ag
	SiGe-aSi/Ag	[3]	40×40	-	8×8[10]	0.01	-	-	~ 50%[11]	3.5/ -	4/0.1	-	BEOL / lift-off / Ag
	WO_x	[4]	11×3	-	11×3	-	1000/~1.8	-	-	0.85/0.05	-	-	SA / lift-off / none
		[5-7]	32×32	-	25×20	~9	>170 / -	-	~ 35%[12]	1.7/ -	3/1[13]	mins to hours @RT[14]	SA / lift-off / none
		[8]	108×54[15]	-	26×10	> 256[16]	-	-	-	-	2.4/1	mins to hours @RT[14]	FI-BEOL / lift-off / none
	Ta_2O_x	[9]	18×2	~100	18×2	-	250 / ~1.1	-	-	-	1500/850	-	SA / lift-off / none
		[10]	16×3	78	4×3	-	1000 / ~2	> 100k	-	1.25/0.1	1800/1300	-	SA / lift-off / none
	TiO_{2-x}	[11,12]	12×12	>50	10×6	0.16	200 / 1.9	>200k	-	0.9/0.17	200/6	>140h@76	SA / lift-off / none
		[13]	20×20	>95	17×20+8×11	0.25	220 /1.5	>100k	< 8%	1.0/0.18	200/6	>20h@120	SA / lift-off / none
		[14]	2×10×10	~100	2×10×10	0.49/2	100/2.5[17]	-	-	1.1/0.15	100/0.1	>25h@100	SA/ion beam milling/ none
		**this work**	**64×64**	**~99**	**64×64**	**0.5625**	**100 / 3.2**	**>100k**	**< 5%**	**1.2/0.13**	**100/6**	**>20h@100**	**SA /RIE/ none**
	HfO_{2-x}	[15]	3D 8×8[18]	-	~120[19]	~1000/8[20]	-	-	binary	-	1200/~300	-	SA / lift-off / none
1T1R	HfO_{2-x}	[16-19]	128×64	>99[21]	128×64	~2500[22]	-	-	<3.1%[23]	2 / -	900/100	10yr @RT	BEOL/lift-off / none
		[20]	128×8	-	960	-	>150 / >3	-	< 35%	-	40/5	-	BEOL/ lift-off / none
		[21]	128×16	>99	128×16	> 5	-	-	3.3 %	-	20/2	~ days @RT	BEOL /lift-off / none
		[22]	158K	-	158 K	1.69	100 / >1.8	< 1k	~ 2-bit	1 / -	10 / 0.1	-	NA / NA / none
		[23]	1K	-	448	~25	-	-	~ 20%	3.5 / -	100/0.1	~1m @30	NA / NA / none

[0] "Crossbar size" refers to the largest-dimension fabricated integrated crossbar circuit (not necessarily fully-functional), while the "largest working demo" refers to the largest number of devices employed at once in the demo, i.e., without relying on post-processing / combining the results from separate measurements. [1] Based on the full pitch of the integrated memory cells. [2] Largest set voltages are used if statistical data are not reported. [3] The test conditions may be different. [4] Specified at 0.1V for the devices with nonlinear static *I-V* characteristics unless noted otherwise. [5] SA = Stand-alone integrated crossbar circuit, RIE = reactive ion etching, BEOL = Back end of line integrated crossbar circuit on CMOS wafer containing access transistors, FI-BEOL = BEOL with fully integrated CMOS peripheral circuits. [6] Denser single devices are reported, though most experimental results are for 25 μm^2 devices. [7] Data for the low-resistance state. Significant retention loss at high resistance levels. [8] Based on Fig. 4c. [9] From Fig. 1d. [10] 40×40 conductance map is based on combining results from separate 25 measurements of 8×8 subarrays. [11] Based on Fig. 4d. [12] Based on Fig. S3 of [5]. Not clear if the data are obtained after tuning all devices or measured immediately after programming each device. [13] Average range of conductance values observed in the crossbar. There is a significant variation between different devices. [14] The effective retention drops with an increase in the utilized conductance range and/or precision of operation - see, e.g., Fig. 6.5 from [30]. [15] 126 6×8 physical subarrays utilized for a logical 108×54 array with the conductances measured after programming each subarray. [16] From Supplementary Note 10. [17] For the top crossbar, while it is 2 V / 50 μA for the bottom one. [18] Effective crossbar dimensions based on 3D-CMOL-like structure (with overlapping electrodes in one direction). [19] Total number of employed devices in one filter based on Fig. 4d. [20] Based on Fig. 2g. Though SEM images of 300-nm-scale devices are shown, all experimental results are based on microscale devices. [21] Based on Fig.1c of [17]. [22] Based on Fig. 1c of [16, 19]. [23] Based on Fig. S3 of [28].

Supplementary Table 2. General circuit and device assumptions

CMOS process feature size	65 nm
Parasitic cap of a minimum metal width[1]	0.21 fF/μm
Parasitic cap of a minimum size thick-oxide device	0.38 fF
Global and local sensing voltage swing	0.15 V
On-resistance of a thick-oxide device	5 kΩ
Minimum area of a thick-oxide device	0.72 μm^2
Minimum area of a thin-oxide device	0.14 μm^2
MIM capacitor	2.5 fF/μm^2

[1] The total parasitic capacitance of electrodes in 0T1R arrays consists of line-to-line capacitance in crossbar structure (M5/M4/M3) that includes coupling and fringing capacitors between conductors (see footnote 3 in Supplimentary Table 3 for more details).

Supplementary Table 3. **Memory assumptions and VMM modeling**. Memory cell assumptions and the detailed breakdown of area and power for 64×64 VMM blocks.

**Memory Cell Assumptions**	0T1R 250nm+500nm (this paper)	0T1R 100nm+150nm (scaled)	1T1R 375 F^2 (scaled)
Cell area (μm^2) [1]	0.75×0.75	0.25×0.25	1.58
G_{on} (μS) [2]	100	16	16
G_{off} (μS) [2]	6.6	1.056	1.056
Cell parasitic capacitance (fF) [3]	0.22	0.055	0.61
Forming / Set / Reset voltage (V) [4]	3.3		
Forming / switching current (μA)	250		

**VMM Characteristics**	0T1R 250nm+500nm (this paper)	0T1R 100nm+150nm (scaled)	1T1R 375 F^2 (scaled)
**General circuit characteristics**			
BL parasitic capacitance of entire channel (fF)	14	3.5	39.56
Maximum output/input current (μA) [5]	100	16	16
**VMM area breakdown (μm^2)**			
A: 64×64 crossbar memory array	2304	256	6471.7
B: Level shifter (per channel) [6]	11.5		
C: Analog programming mux (per channel) [8]	10.5	6.375	7.13
D: Local sensing circuit (per channel) [7]	63.8	50.35	50.35
E: 6×64 decoder	750		
F: Buffered 4-bit current-steering DAC (per channel)	88.8	75.3	75.3
Total single-ended 64×64 analog VMM [9]	8395	7010	13579
Total single-ended 64×64 mixed-signal VMM [10]	11237	9421	15991
Global sensing circuit (per channel) [11]	113.8	100.35	100.35
**VMM power breakdown (μW)**			
Average crossbar power (per channel) [12]	60	19.2	19.2
Local sensing (per channel) [12]	17.2	11.02	11.02
Buffered 4-bit current-steering DAC (per channel)	37.74	22.08	22.08
Global sensing [11]	35.2	31.9	31.9

[1] For the demonstrated 0T1R crossbar, the electrode width is 250 nm, and the gap size is 500 nm. In the case of scaled 0T1R technology, 100×100 nm^2 cell footprint and 150 nm spacing between metal lines are based on design rules for the considered 65 nm process. The assumptions of 1T1R memory are somewhat aggressive when compared to the state-of-the-art demonstrations (Supplementary Table 1). For example, in analog-grade 1T1R device technology [16-19], the cell size is 2,500 μm^2, while switching currents and midrange conductance are ~10× higher. ~100 F^2 cell area in F = 22 nm FinFET technology, which is equivalent to ~0.42 μm^2 in 65 nm planar CMOS process, and 50 μS midrange conductance were reported in [24].

[2] Device conductance is assumed to scale linearly with the device footprint [13].

[3] $C_{0T1R} = (C_{A\_BOT}w + 2C_{F\_BOT} + C_{A\_TOP}w + 2C_{F\_TOP} + 2C_{Fring})(w + g)$ and $C_{1T1R} = C_{0T1R} + C_{diff}$, where w is the width of the electrode, g is the gap size between electrodes, and C_{diff} is the diffusion capacitance of the selector in the ohmic regime, and other parameters are obtained from the process design kit, i.e. C_{A_BOT} = 0.24 fF/μm^2, C_{A_TOP} = 0.24 fF/μm^2, C_{F_BOT} = 1.07×10^{-2} fF/μm, C_{F_TOP} = 1.1×10^{-2} fF/μm, C_{Fring} = 6.5×10^{-2} fF/μm.

[4] The maximum input voltage drop during forming of the device is 4 V, from which 3.3 V is dropped on a memristor and 0.7 V on analog programming muxes. All programming switching is designed using thick-oxide MOSFETs.

[5] The maximum input and output currents were found to be $<10I_{max,cell}$ from the detailed kernel mapping to 64×64 VMM blocks for representative neural networks [26].

[6] Level shifters are used to translate the output voltage from 1.2 V decoders to 3.3 V programming switches.

[7] Local sensing circuitry is optimized according to the VMM block parasitics [25,26].

[8] The sizing of analog switches is obtained according to the caption of Supplementary Figure 11.

[9] The total area for analog VMM block is calculated as max[A, 128(B+C) + 64D + 2E] and A + 128(B+C) + 64D + 2E for 0T1R and 1T1R implementations, correspondingly. The max is due to the assumption of overlap between peripheral circuits and memory.

[10] The total area for mixed-signal VMM is calculated as max[A, 128(B+C) + 64D + 2E + 32F] and A + 128(B+C) + 64D + 2E + 32F for 0T1R and 1T1R implementations, correspondingly. The factor of 32 is due to using a 4-bit differential DAC circuit.

[11] Figure 5a explains the distributed local/global sensing implementation.

[12] The estimates are for one output channel so that the total for the 64-output VMM circuit is 64 times larger.

Supplementary Table 4. Performance estimates for the two studied applications.

7-layer fully analog MLP [1]	0T1R 250nm+500nm (this work)	0T1R 100nm+150nm (scaled)	1T1R 375 F^2 (scaled)
Total number of registers & register area (μm^2) [2]	4096 & 21		
Total number of 64×64 blocks	51344		
2% settling time (ns)	42.7	54.8	78.3
Total power (W)	60.55	37.55	37.55
Throughput (Mfps & POp/s)	23.4 & 4.92	18.2 & 3.8	12.77 & 2.68
Total area (cm^2)	4.34	3.62	6.99
Memory efficiency (%) [3]	27.25	3.6	47.47
Energy per frame (μJ/frame)	2.58	2.05	2.94
Energy efficiency (TOp/J)	81.33	102.1	71.51

4-bit aCortex accelerator with external DACs [4]	0T1R 350nm+350nm (this work)	0T1R 100nm+150nm (scaled)	1T1R 375 F^2 (scaled)
Total power (mW)	683	367	394
Throughput (Kfps & TOp/s)	3.88 & 10.44	4 & 10.5	3.41 & 9.18
Total area (mm^2)	980	832	1370
Memory efficiency (%) [3]	19.24	2.52	38.69
Energy per frame (μJ/frame)	175	93	115
Energy efficiency (TOp/J)	15.2	28.84	23.3

[1] Fully-analog 7-layer (1024-16384-4096-4096-1024-256-100) MLP circuit consists of ~105M weights and utilizes architecture similar to the one described in Fig. 5a. 4-bit buffered current steering DACs are assumed in the front-end of the network. The neurons in the last layer are assumed to be loaded with a 1 pF capacitor.

[2] Registers are required for buffering input data in the MLP circuit.

[3] Memory efficiency is reported as a fraction of the area occupied by memory cells. In the case of 0T1R circuits, memory cell arrays are overlapped with peripheral circuits.

[4] We assume 4-bit aCortex architecture [26] utilizing mixed-signal 64×64 VMM blocks with 4-bit buffered current steering DACs. The performance is evaluated for Google's deep recurrent network for language translation (GNMT) benchmark with ~134 M weights.

Supplementary Note 1: Phenomenological Dynamic Model

The main purpose of the model is to estimate the change in device conductance ΔG, with respect to the initial conductance G_0, all measured at small non-disturbing (read) voltage 0.1 V, upon application of write voltage pulse with amplitude V and a fixed duration of 2 ms. The fixed duration is assumed for simplicity, i.e., to avoid explicit dependence of conductance change on pulse duration in the model. This simplification is also justified because of a similar fixed-duration pulse approach utilized in the tuning algorithms. (In a more advanced algorithm, variable time duration could be used for faster convergence [27]). Because of the long memory state retention for the developed metal-oxide memristors, i.e., their strongly nonlinear switching kinetics, obtaining meaningful experimental data for fitting conductance changes at half of the nominal write voltages required applying very long, with up to 2 ms duration pulses (Supplementary Figure 7a-d). This is the main difference compared to the phenomenological model presented in Ref. 28, which used experimental data for a narrower range of write voltage pulse amplitudes and durations to derive dynamic model, and hence somewhat inaccurate in predicting conductance changes at smaller, half-bias voltages. The following function is found to fit well experimental data for both set and reset switching

$$\frac{\Delta G}{G_0} \approx \exp\left[\frac{\beta_1}{1+\beta_2(\alpha V)^2}\right] \sinh\left[\beta_3 \frac{\alpha V}{1+\beta_2(\alpha V)^2}\right] \left(\gamma_1 + \gamma_2\sqrt{G_0} + \gamma_3 G_0\right), \quad \text{(S1)}$$

where $\beta_1, \beta_2, \beta_3, \gamma_1, \gamma_2$ and γ_3 are fitting parameters common for all devices (Supplementary Figure 7g), while α is a unique scaling parameter for each device that represents device to device variations in the switching threshold (Supplementary Figure 7h).

Specifically, the model for the average behavior, with fixed $\alpha = 1$, is first found by fitting a surface to the experimental data for the average conductance changes, i.e. $\{<\Delta G/G_0>, G_0, V\}$ data points (Supplementary Figure 7e,f).

As a reminder, the effective set (reset) switching threshold of the crossbar array is defined as a voltage at which the small-voltage conductance is changed from its extreme value G_0 = 14 μS (75 μS) by more than 20%, i.e., $|\Delta G|/G_0 = 0.2$ when applying increasing amplitude positive (negative) voltage ramp (Fig. 2e). According to the fitted model, $V_{set}^* = 1$ V and $V_{reset}^* =$ -1.4 V for $\alpha = 1$. The experimentally measured threshold voltages (Fig. 2f) are well approximated with log-normal distributions with parameters $\mu = 0.14$ and $\sigma = 0.25$, and $\mu = 0.29$ and $\sigma = 0.26$ for set and reset switching, respectively. According to the selected fitting function, parameter α is a multiplicative factor for the applied voltages. Hence, when modeling distribution in set threshold voltages of a crossbar circuit, we first randomly initialize V_{set} for each crosspoint device by sampling it from the fitted set threshold log-normal distribution and then find the corresponding $\alpha_{set} = V_{set}^*/ V_{set}$. A similar approach is used to initialize α_{reset}. An example of the generated α using such approach and corresponding threshold voltages predicted by the model are shown in Supplementary Figure 7h and 7i, respectively.

Finally, since the experimentally observed variations in set and reset threshold voltages (i.e. the relative standard deviations or the coefficient of variations) are very similar, for simplicity, we use the same α when sweeping variations in the modeling studies (Fig. 5 and Supplementary Figure 9).

Supplementary Note 2: System-Level Performance Estimates

To demonstrate the prospects of 0T1R technology, we model the performance of two representative neuromorphic architectures - aCortex [26], which is an energy-efficiency-optimized multi-purpose architecture for the acceleration of a wide range of neural network inference models, and a fully-analog large-scale (1024-16384-4096-4096-1024-256-100) multilayer perceptron with ~105M parameters, which is especially suitable for high-throughput inference tasks. All peripheral circuits, digital blocks, and circuits for conductance tuning are designed in the 65 nm CMOS process – see Supplementary Table 2-4 for more details. The performance is evaluated using the results of physical layout and SPICE simulations of the major components. All designs involve 64×64 physical crossbar circuits, while differential implementation based on two physical crossbar circuits, i.e., similar to the architecture shown in Fig. 5a, are assumed for 64×64 VMM operation. The details of the simulation methodology for aCortex were presented in [26]. For MLP, the complete signal path from the network's input to the output is properly modeled by simulating signal propagation in VMM blocks and taking into account the intra-block parasitic capacitance of the global lines. Furthermore, three technology options are evaluated – the one with parameters close to the demonstrate device, 65-nm 0T1R, and 65-nm 1T1R devices with the parameters shown in Supplementary Table 2b.

The simulation results for the fully-analog MLP implementation with the demonstrated technology show 5.61 μJ/f energy-efficiency and 16.6 Mf/s throughput with 12.95% of 4.07 cm^2 of the chip occupied by the memristors. When scaled down to 65 nm, though throughput reduces due to 11.6 Mf/s, the overall energy efficiency improves because of power scaling in the array and amplifiers. As expected, the area slightly improves to 3.17 cm^2 for 65-nm 0T1R technology, though it becomes substantially larger (increased to 6.39 cm^2) for 1T1R technology.

The simulation results for aCortex show that the inference time for GNMT benchmark tasks slightly improves when scaling 0T1R technology. Though peripheral circuits become slower in scaled 0T1R circuits due to the reduced midrange device conductance, the upshot is a more compact implementation of VMM blocks, which results in less parasitics in the digital circuits and much faster data transfer. Most importantly, the density of the scaled 0T1R aCortex chip is better by a factor of ~18×, while throughput and energy efficiency also substantially higher compared to the 65-nm 1T1R design.

Our estimates show that further scaling down of the technology will increase the gap between passive and active memories even more if the switching voltage and currents remain the same. Alternatively, with appropriate scaling of cell currents, more efficient and compact peripheral circuits can be utilized to improve memory efficiency, especially in the MLP circuit, potentially matching that of embedded NOR flash memory aCortex [26]. Furthermore, the memory efficiency is expected to improve significantly with periphery sharing and 3D memory integration.

References

[1] Strukov, D. B. Tightening grip. *Nat. Mater.* **17**, 293-295 (2018).

[2] Yeon, H. *et al.* Alloying conducting channels for reliable neuromorphic computing. *Nat. Nanotechnol.* **15**, 574-579 (2020).

[3] Kim, K. *et al.* A functional hybrid memristor crossbar-array/CMOS system for data storage and neuromorphic applications. *Nano Lett.* **12**, 389-395 (2011).

[4] Shin, J. *et al.* Hardware acceleration of simulated annealing of spin glass by RRAM crossbar array. in *IEEE International Electron Device Meeting (IEDM),* 18.63-18.64. (IEEE, 2018).

[5] Sheridan, P. M. *et al.* Sparse coding with memristor networks. *Nat. Nanotechnol.* **12**, 784 (2017).

[6] Moon, J. *et al.* Temporal data classification and forecasting using a memristor-based reservoir computing system. *Nat. Electron.* **2**, 480-487 (2019).

[7] Ma, W. *et al.* Device nonideality effects on image reconstruction using memristor arrays. in *IEEE International Electron Device Meeting (IEDM)*, 16.7.1-16.7.4. (IEEE, 2018).

[8] Cai, F. *et al.* A fully integrated reprogrammable memristor–CMOS system for efficient multiply-accumulate operations. *Nat. Electron.* **2**, 290-299 (2019).

[9] Choi, S. *et al.* Experimental demonstration of feature extraction and dimensionality reduction using memristor networks. *Nano Lett.* **17**, 3113-3118 (2017).

[10] Jeong Y. *et al.* K-means data clustering with memristor networks. *Nano Lett.* **18**, 4447-4453 (2018).

[11] Prezioso, M. *et al.* Modelling and implementation of firing-rate neuromorphic-network classifiers with bilayer Pt/Al_2O_3/TiO_{2-x}/Pt memristors. in *Proc. IEEE International Electron Device Meeting (IEDM),* 17.4.1-17.4.4. (IEEE, 2015)

[12] Prezioso, M. *et al.* Training and operation of an integrated neuromorphic network based on metal-oxide memristors. *Nature* **521**, 61 (2015).

[13] Merrikh Bayat, F. *et al.,* Implementation of multilayer perceptron network with highly uniform passive memristive crossbar circuits. *Nat. Commun.* **9**, 2331 (2018).

[14] Adam, G. *et al.* 3-D memristor crossbars for analog and neuromorphic computing applications. *IEEE Trans. Electron Devices* **64**, 312-318 (2016).

[15] Lin, P. *et al.* Three-dimensional memristor circuits as complex neural networks. *Nat. Electron.* (2020).

[16] Li, C. *et al.* Efficient and self-adaptive in-situ learning in multilayer memristor neural networks *Nat. Commun.* 9, 2385 (2018).

[17] Hu, M. *et al.* Memristor-based analog computation and neural network classification with a dot product engine. *Adv. Mat.* **30**, 1705914 (2018).

[18] Li, C. *et al.* Long short-term memory networks in memristor crossbar arrays. *Nat. Mach. Intell.* **1**, 49 (2019).

[19] Wang, Z. *et al.* Reinforcement learning with analogue memristor arrays. *Nat. Electron.* **2**, 115 (2019).

[20] Yao, P. *et al.* Face classification using electronic synapses. *Nat. Commun.* **8**, 15199 (2017).

[21] Yao, P. et al. Fully hardware-implemented memristor convolutional neural network. *Nature* **577**, 641-646 (2020).

[22] Liu, Q. *et al.* A fully integrated analog ReRAM based 78.4 TOps/W compute-in-memory chip with fully parallel MAC computing. in *Proc. IEEE International Solid-State Circuits Conference (ISSCC)*, 500-501. (IEEE, 2020).

[23] Zheng, X. *et al.* Error-resilient analog image storage and compression with analog-valued RRAM arrays: An adaptive joint source-channel coding approach. in *Proc. IEEE International Electron Device Meeting (IEDM),* 3.5.1-3.5.4. (IEEE, 2018).

[24] Golonzka, O. *et al.* Non-volatile RRAM embedded into 22FFL FinFET technology. in *Proc. Very Large Scale Integration Symposium (VLSISymp)*, T230-231. (IEEE, 2019).

[25] Mahmoodi, M.R & Strukov, D. An ultra-low energy internally analog, externally digital vector-matrix multiplier based on NOR flash memory technology. in *Proc. Design Automation Conference (DAC)*, 1-6. (ACM/ESDA/IEEE, 2018).

[26] Bavandpour, M., Mahmoodi, M.R., & Strukov, D.B. aCortex: An energy-efficient multi-purpose mixed-signal inference accelerator. *IEEE J. Explor. Solid-State Computat.* **6**, 98–106 (2020).

[27] Merrikh Bayat, F. *et al.* Model-based high-precision tuning of NOR flash memory cells for analog computing applications. in *Proc. Device Research Conference (DRC)*, 1-2. (2017).

[28] Nili, H. *et al.* Comprehensive compact phenomenological modeling of integrated metal-oxide memristors. *IEEE Trans. Nanotechnol.* **19**, 344-349 (2020).

[29] Prezioso, M., Merrikh-Bayat, F., Chakrabarti, B. & Strukov, D.B. RRAM-based hardware implementations of artificial neural networks: Progress update and challenges ahead. in *Proc. SPIE'16 Photonics West*, art. 974918 (SPIE, 2016).

[30] Jo, S. H. Nanoscale Memristive Devices for Memory and Logic Applications. University of Michigan, Ph.D. Dissertation, 2010.
